# Cryotherapy for nodal metastasis in NSCLC with acquired resistance to immunotherapy

**DOI:** 10.1186/s40425-018-0468-x

**Published:** 2018-12-12

**Authors:** Lucas C. Adam, Junaid Raja, Johannes M. Ludwig, Adebowale Adeniran, Scott N. Gettinger, Hyun S. Kim

**Affiliations:** 1 Division of Interventional Radiology, Department of Radiology and Biomedical Imaging, Yale School of Medicine, 330 Cedar Street, New Haven, CT 06510 USA; 2 Department of Diagnostic and Interventional Radiology and Neuroradiology, University Hospital Essen, University of Duisburg-Essen, Hufelandstr. 55, 45147 Essen, Germany; 3 Department of Pathology, Yale School of Medicine, 330 Cedar Street, New Haven, CT 06510 USA; 4 Division of Medical Oncology, Department of Medicine, Yale School of Medicine, 330 Cedar Street, New Haven, CT 06510 USA; 5 Yale Cancer Center, Yale School of Medicine, 330 Cedar Street, TE 2-224, New Haven, CT 06510 USA

**Keywords:** NSCLC, Acquired resistance, Immune checkpoint inhibitors, Cryoablation

## Abstract

Novel approaches with checkpoint inhibitors in immunotherapy continue to be essential in the treatment of non-small cell lung cancer (NSCLC). However, the low rate of primary response and the development of acquired resistance during the immunotherapy limit their long-term effectiveness. The underlying cause of acquired resistance is poorly understood; potential management strategies for patients with acquired resistance are even less clear. Here, we report the case of a 75-year-old female smoker with cough, fatigue, and weight loss that was found to have an 8.6 cm right upper lobe lung lesion with local invasion, adenopathy, and a malignant pericardial effusion. This lesion was biopsied and identified to be cT3N3M1b squamous cell cancer of the lung without any recognizable PD-L1 expression on tumor cells. For her metastatic NSCLC, the patient underwent two lines of conventional chemotherapy before initiation of combination immunotherapy with an anti-PD-L1 and anti-CTLA-4 antibody. Though she initially achieved a response, she thereafter progressed and developed immunotherapy resistant lymph nodal metastasis. While cervical lymph nodes could be surgically removed, another metastasis in an aortocaval area required a more sensitive therapy like thermal ablation. The aortocaval node was partially treated with a single treatment of cryotherapy and demonstrated durable complete response. Cryotherapy for checkpoint immunotherapy resistant metastasis appears to be a safe and feasible treatment for treating metastatic disease in non-small cell lung cancer. The prospect of cryotherapy adjuvancy may enable local control of metastatic disease after initial response to immune checkpoint immunotherapy and may impact on overall outcomes.

## Case presentation

**Fig. 1:**
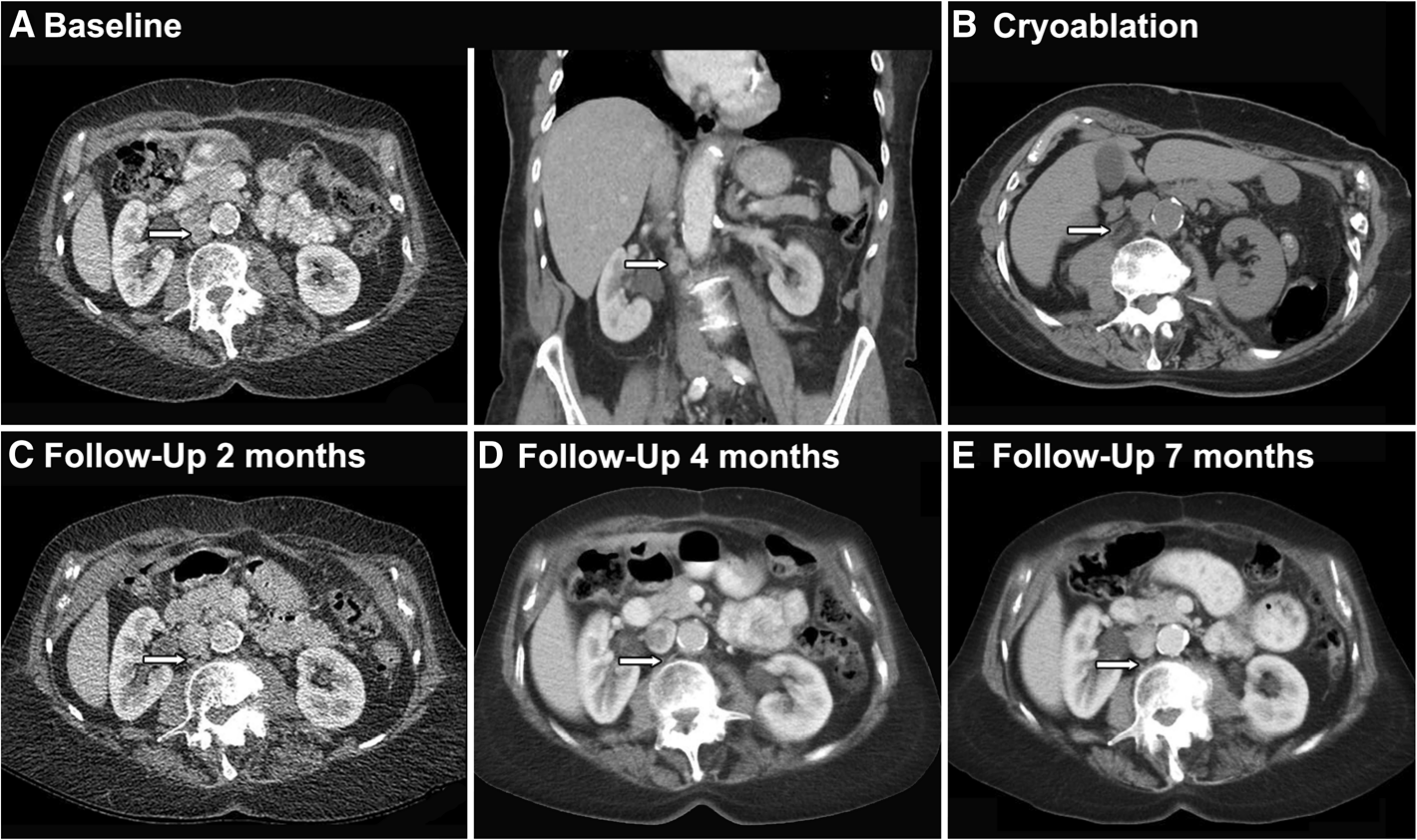
Imaging of patient undergoing combination anti-PD-L1 and anti-CTLA-4 immunotherapy. **a** Baseline CT of the abdomen demonstrated an aortocaval lymph node. **b** Axial CT obtained during cryoablation demonstrates an ice ball. **c - e** Follow-up CTs showing completely responsive lymph node after partial cryotherapy

**Fig. 2:**
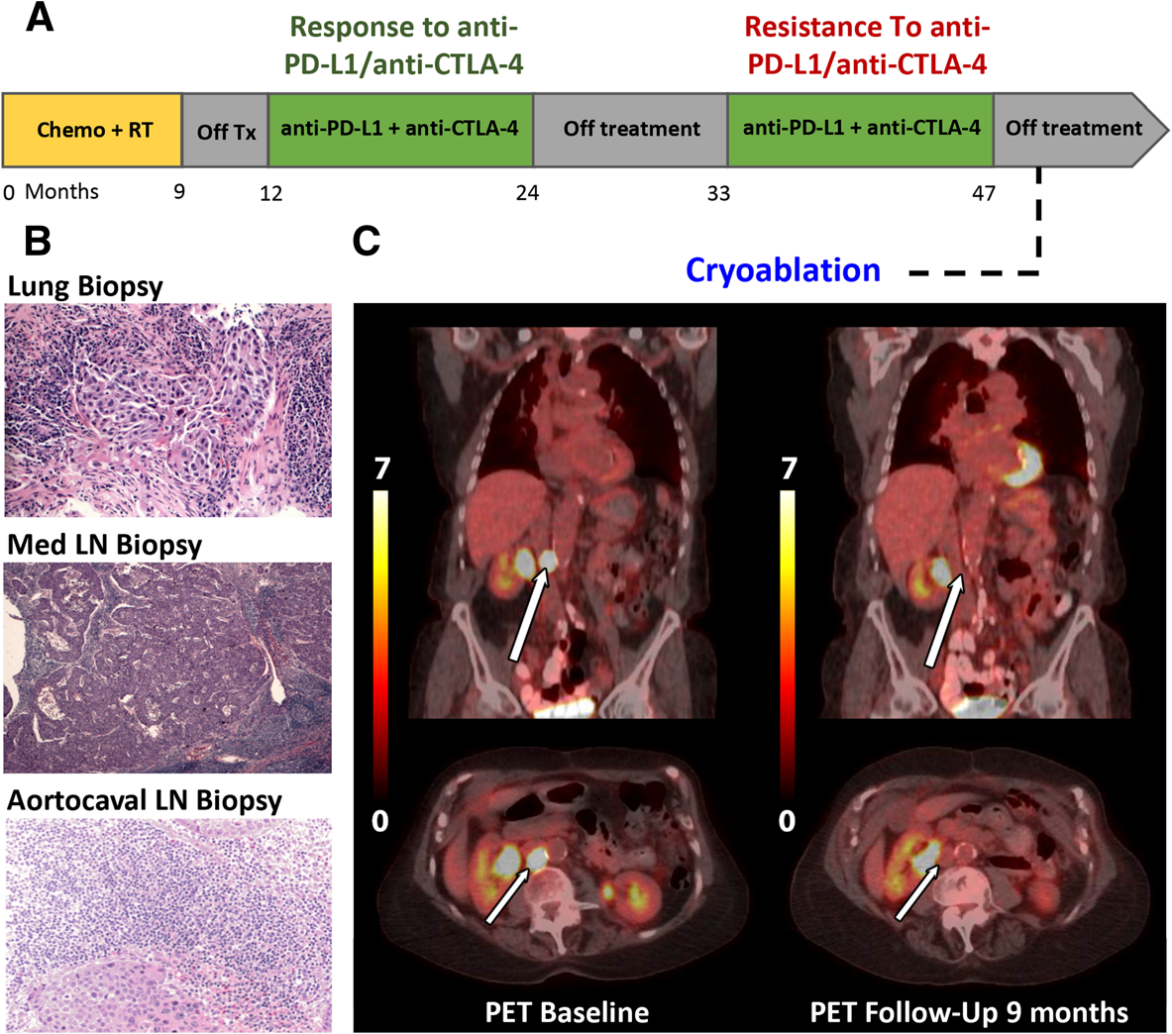
Cryoablation of an aortocaval lymph node in the setting of acquired resistance during immune checkpoint inhibition. **a** Clinical timeline of therapy. **b** Poorly differentiated squamous cell carcinoma in lung tissue, mediastinal lymph node, and ablated aortocaval lymph node (H&E, original magnification X200) before cryoablation. **c** Baseline 18FDG PET/CT was obtained two months prior to cryoablation. Nine months follow-up PET imaging shows no recurrence of the metastatic lymph node

A 75-year-old female former smoker with a 30 pack-year history initially presented with fatigue, cough, and weight loss. She underwent a chest CT that demonstrated an 8.6 cm right upper lobe lesion with mediastinal invasion, extensive cervical and mediastinal adenopathy, and a malignant pericardial effusion (Fig. 1a). Upon biopsy this was proven to be squamous cell carcinoma that was TTF-1/NAPSIN negative and was staged as stage IV cT3N3M1b. Subsequently she developed hypercalcemia as a paraneoplastic complication. She was then initiated on palliative PT-DC with carboplatin and gemcitabine and then maintenance gemcitabine with a transient partial response. Thereafter she underwent palliative radiotherapy to her right lung, and subsequent salvage chemotherapy with docetaxel without response. She was then started on combination immunotherapy with PD-L1 and CTLA-4 antibody therapy. Histopathology did not reveal any PD-L1 expression on tumor cells prior to immune therapy initiation. After initial partial response, she developed oligo-progressive disease in a celiac lymph node that was resected with continuation of immunotherapy. Treatment holiday was initiated one year after starting combination immunotherapy, at which point no active disease was appreciated on imaging. Approximately nine months thereafter she was noted to have recurrence of disease and resumed the same combination immunotherapy for an additional one year. There was initially complete response during this course; however, the final staging study demonstrated focal progression of disease with multiple new cervical lymph nodes and a new aortocaval lymph node (Fig. 2).

Tissues from the cervical lymph node and aortocaval lymph node biopsy confirmed poorly-differentiated squamous cell carcinoma consistent with her known lung cancer (Fig. 2b). The cervical lymph nodes were surgically excised. Given the sensitive location of the abdominal lymph node, image guided locoregional therapy was planned. The cryoablation was then performed on the aortocaval lymph node with complete response on follow-up imaging (Fig. 1b). Subsequent serial imaging showed durable complete control at the local aortocaval lymph node site for at least nine months with no additional oncologic therapy (Fig. 1c-e).

## Discussion

In recent years immune checkpoint inhibitors targeting the PD-1 axis have emerged as effective therapies for multiple malignancies. For NSCLC, the majority of patients who do achieve initial objective response will ultimately acquire treatment resistance to immune checkpoint blockade with tumor progression [1]. The underlying mechanisms of primary and acquired resistance to PD-1 axis inhibitor therapy in NSCLC are not fully understood. One potential approach to overcome such resistance is to use locoregional therapy as an adjuvant to immune checkpoint inhibitor therapy to locally control disease and to potentially enhance immune priming. Here, we report a case of successfully performing cryoablation with sustained complete response in a metastatic lymph node after acquired treatment resistance to anti-PD-L1 and anti-CTLA-4 immune therapy in NSCLC.

There are limited publications on safety and feasibility of combining cryoablation and immune therapy in cancer as well as molecular mechanisms of potential synergistic treatment effects. Several pre-clinical research studies have demonstrated that combining cryoablation and checkpoint inhibitors can lead to synergistic immune responses in cancer [2]. Clinically, this promising treatment strategy has not yet been formally established. To date, the potential effect of cryoablation and checkpoint inhibition for NSCLC are unknown and there are two phase II trials planned that aim for new insights into this therapy combination in lung cancer (ClinicalTrials.gov identifier: NCT03290677 and NCT02469701).

Two recent studies in other solid tumors have provided insights into the feasibility of cryo-immuno treatments. The study by Duffy et al. enrolled 11 hepatocellular carcinoma (HCC) patients in a setting in which patients received cryoablation in addition to tremelimumab, a CTLA-4 inhibitor. The combination therapy proved to be safe and it was further noted that some patients accumulated CD8+ T- cells in tumor tissue and thereby potentially enhanced the therapeutic effect [3]. The study by McArthur et al. aimed to determine the feasibility of combining cryoablation with ipilimumab, another CTLA-4 inhibitor, in breast cancer and was found to be safe and also trigger a systemic immune reaction in patients [4]. A study on renal cell carcinoma (RCC) metastatic to the lung further demonstrated cryoablation combined with an immune stimulatory drug is also practicable and safe for lung metastases [5].

Identifying the mechanisms that lead to resistance as treatment progresses is one of the critical challenges in immunotherapy [6]. Higher mutation-associated neo-antigen load bears an increased likelihood of immune response, thus, elevated chance of successful checkpoint inhibition. Recently the mutagens of pretreated NSCLC with post-PD-1-therapy tumor cells were investigated with a loss of 7 to 18 putative mutation-associated neo-antigens in tumors of patients who acquired therapy resistance [7]. The selective pressure of immunotherapy seemingly removed cancer cell neo-antigens of close MHC binding affinity, leading to reduced T-cell recognition. Additional mechanisms of immune escape may be found in the adaption of the tumor microenvironment and upregulation of alternative immune checkpoints [8]. Future therapies may aim at increasing the exposure of tumor neo-antigens, and thus, reinduce an endogenous immune response. The combination of checkpoint inhibitors and locoregional therapies could provide one possibility to increase tumor neo-antigen recognition by immune cells [9].

When surgical resection is not feasible, radiation therapy and thermal ablation remain as viable alternatives for patients. Both therapy options are broadly used as monotherapies in NSCLC patients. Although they have comparable clinical outcomes, they differ in number of required treatments, treatment costs, and complications [10]. At this point, there are ongoing clinical trials investigating concordant or sequential application of immunotherapy and radiation therapy. Preliminary data suggests a feasible, and maybe superior effectivity when used combined in NSCLC patients rather than alone [11, 12]. Similarly, the combination of thermal ablation and simultaneous immune therapy seems to positively affect therapeutic outcome, suggesting an amplification of immune response. Thermal ablation is routinely used in the clinic and established as a safe and feasible alternative for NSCLC Stage I and II patients not eligible for surgical resection [13]. Current thermal ablative techniques such as cryoablation, radiofrequency ablation (RFA), microwave ablation (MWA), and focused ultrasound (FUS) have all been shown to induce at least a partial destruction of tumor tissue, and, possibly involving a systemic antitumor immune response. In order to activate the acquired immune system, locoregional ablation needs to trigger antigen presentation and recognition by T-cells, interaction of costimulatory signals and presence of danger signals [14]. In preclinical and clinical data, immune interactions were especially apparent in RFA and cryoablation with lower immune modulatory effects seen for FUS and MWA [15]. In contrast to hyperthermia, freezing prevents the destruction of protein ultrastructures and causes increasing permeability of plasma membranes of tumor tissue. In consequence, pro-inflammatory cytokines and intratumoral cell fragments such as organelles and damage associated molecular patterns (DAMPs) are released in the proximate blood stream. By phagocytizing these DAMPs, dendritic cells (DCs) express co-stimulatory CD80/86 molecules and thereby promote T-cell immune response [16]. This makes cryoablation an intriguing technique to amplify an immune response by checkpoint inhibitors in patients of NSCLC.

Combined cryoablation with anti-CTLA-4 therapy identified a significant increase in effector T-cells in ablated tissue. Findings of an animal study on prostate cancer confirmed that cryoablation combined with immunotherapy is more effective than immunotherapy alone. Beyond the effective reduction of the primary tumor, a second tumor that was implanted one day post-cryoablation showed a strong CD8+ T-cell response [17]. This systemic tumor immunization may have compensated the initial loss of specific antigen immune response that is affiliated with acquired resistance. The same synergistic effect of immunotherapy and cryoablation in overcoming treatment resistance potentially led to disease stabilization in our patient.

**Fig. 3:**
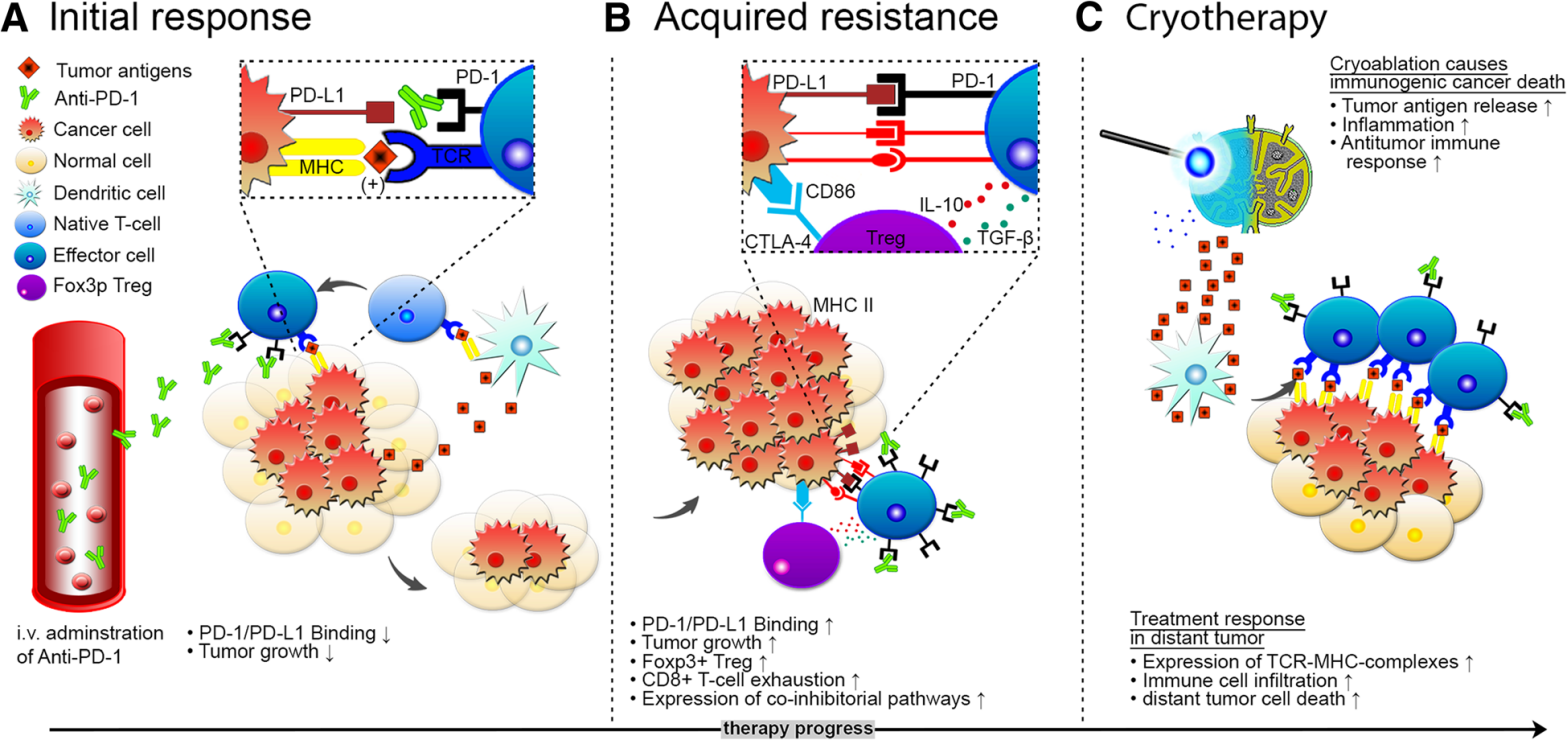
Hypothesis of overcoming acquired resistance. Cryoablation may have boosted immune response in acquired resistance of anti-PD-1 axis therapy for NSCLC when concomitantly used with a PD-1 inhibitor. **a** Anti-PD-1 therapy blocks inhibiting pathways and increases T-cell response. **b** CD8+ T-cell exhaustion, upregulation of alternative co-inhibitory immune pathways. **c** Partial cryoablation of a lymph node increases dendritic cell load and boosts T-cell response against tumor cells in residual lymph node tissue and maybe also in distant tumor

It could be hypothesized that the ablated lymph node in our patient may have led to a prompt increase in dendritic cell load of tumor antigens and induced a T-cell immune response leading to locoregional control of the cancerous lymph node (Fig. 3). On the HCC tumor microenvironment, it has been shown that PD-L1 circulation and intratumoral PD-L1 expression significantly increases one week after cryoablation [18]. This mechanism could reinforce treatment efficacy when performed simultaneously to systemic PD-1 inhibitor therapy. Yet, the effect of cryoablation on PD-1 expression needs further investigations in NSCLC. Further studies are required to determine the cryoablation effects in the reversal of immune cell exhaustion during checkpoint therapy.

Overall, the findings of this case indicate that cryotherapy for the acquired resistance to PD-1/PD-L1 and CTLA-4 axes immunotherapy for local control of NSCLC may provide a successful, safe and feasible strategy to complement immunotherapy in local control, bridging between systemic therapies and potentially overcoming the acquired resistance to checkpoint inhibition immunotherapies.

## Abbreviations

CT: Computer tomography
CTLA-4: Cytotoxic T-lymphocyte-associated protein 4
DAMPs: Damage associated molecular patterns
DC: Dendritic cell
FUS: Focused ultrasound
HCC: Hepatocellular carcinoma
MWA: Microwave ablation
NSCLC: Non-small-cell lung carcinoma
PD-1: Programmed cell death protein 1
PT-DC: Platinum-based doublet chemotherapy
RCC: Renal cell carcinoma
RFA: Radiofrequency ablation
